# Physical Therapeutics

**Published:** 1900-12-08

**Authors:** 


### PHYSICAL THERAPEUTICS.

The establishment of a journal devoted to physical therapeutics seems to suggest that in the future much more attention will be paid to this branch of medicine than has been the case in the past, although it is, perhaps, to be regretted that anything should occur to make people regard the measures included under this term as a specialty apart from ordinary practice.

We are all ready enough to laugh at the pompous old gentleman who plays the part of the physician in the older novels and story books. Nevertheless, we may express our belief that we have not even yet shaken ourselves free of some of the evils and some of the prejudices which date back to what may be called the gold-headed-cane period of medicine, one of the worst of which is the tendency to confine our therapeutic efforts to pills and mixtures, instead of using every means which experience, and not merely the experience of the schools, shows to be of service. So great, however, is the faith of the public in the ever-present bottle of physic that if a medical man attempts to personally look after the treatment of his patient, to do a little massage, to apply resistance exercises, or to look after the giving of the bath, there are plenty of people—and among his professional brethren there are any number—who are quite ready to say, "Yes Mr. A is a very good fellow, most kind and attentive, but is he not a bit of an old woman?" Experience teaches that more credit is gained and less responsibility is undertaken by ordering some medicine and leaving the patient to fight it out as best he may than by personally undertaking physical treatment. The patient feels that the practitioner is acting "just like a London physician," and what more can anyone desire? So the physic bottle and the pill box still reign in the land, as the sign and the emblem of orthodox medicine; all of which is very wrong.

Disease may be treated in several ways. First, there is the extremely ignorant, totally empirical, but sometimes extraordinarily successful method, which we may call the alterative plan; a method allied to the common trick of giving one's watch a good shake if it does not go. One gives the poor man a strong pill, or a Turkish bath; if he is fat one starves him, or if thin one gives him a course of overfeeding; while a lazy man one sets upon a bicycle with thirty miles a day before him. Any way, one gives him a good shake up, and so great is man's vitality that very often he is all the better for it. Of course we have no sympathy with this sort of treatment, notwithstanding the fortunes which come to those who carry it out in an impressive manner. Then there is the treatment which depends for its success upon the fact that many of our diseases are due to toxic materials of one sort and another, and that some of these materials can be either neutralised or removed by drugs. This is at least "scientific." It fairly smells of the test tube and the laboratory; and, moreover, it is easy, for difficult as it must be to discover a real antidote to these various hypothetical toxins, it is by no means difficult to persuade both oneself and the patient that one has got hold of the very thing. Then there are many other methods according to which bottles of medicine may be brought to bear upon disease, and lastly, there is the line of treatment which aims at removing the toxins, at sweeping out the "effete materials" by which disease is caused, and at "stimulating normal function," partly by altering the physical environment, for example, by the application of heat, light, electricity, water, vapour, &c., and partly by the use of movements and exercises, by aid of which the different parts of the body are made to react upon, or "stimulate" each other. Such physical therapeutics, however, mostly require apparatus, and so smell of quackery —such are the restrictions imposed upon our practice by the gold-headed-cane ideals of medical life—or they are suggestive of masseuses, medical rubbers, and bathmen, people with whom etiquette and even some dicta of the General Medical Council warn us to form no alliances. Hence, unless particular methods of physical treatment have become crystallised into fixed routines, so that they can be prescribed *en bloc*, they are made but little use of. At a number of places certain special methods of treatment are adopted which are as easy to order as a pill—and so they are ordered. Nothing is more simple, and professional withal, than to prescribe a course at Nauheim, or Aix-la-Chapelle, or Droitwich. But to superintend the exercises or the baths or the inunctions, and so to cure one's patient one's self, is quite another thing. First of all it is a good deal of trouble, but above all it is a little outside ordinary professional usage. We cannot but think, however, that English medical men will before long find out the advantage of taking under their own charge the whole treatment of disease, and not contenting themselves with the comparatively small part which is involved in prescribing what should be done.

